# Supplementary material for: Modeling glioblastoma heterogeneity as a dynamic network of cell states
Source: Mol Syst Biol. 2021 Sep 16;17(9):e10105. doi: 10.15252/msb.202010105 (PMC8444284; doi:10.15252/msb.202010105)
Supplement: Supplementary file 5 — Source Data for Figure 3 [file MSB-17-e10105-s001.zip › Figure3A_sourcedata/GSEA_3065/hallmarks_state3.GseaPreranked.1623416274744/HALLMARK_MYC_TARGETS_V1.html]

Details for gene set HALLMARK\_MYC\_TARGETS\_V1[GSEA]

|  || Dataset | state3 |
| Phenotype | NoPhenotypeAvailable |
| Upregulated in class | na\_neg |
| GeneSet | HALLMARK\_MYC\_TARGETS\_V1 |
| Enrichment Score (ES) | -0.5009425 |
| Normalized Enrichment Score (NES) | -1.9501573 |
| Nominal p-value | 0.0 |
| FDR q-value | 2.6666667E-4 |
| FWER p-Value | 0.001 |
Table: GSEA Results Summary

  

Fig 1: Enrichment plot: HALLMARK\_MYC\_TARGETS\_V1      
 Profile of the Running ES Score & Positions of GeneSet Members on the Rank Ordered List

  

| PROBE | GENE SYMBOL | GENE\_TITLE | RANK IN GENE LIST | RANK METRIC SCORE | RUNNING ES | CORE ENRICHMENT || 1 | CANX |  |  | 27 | 0.479 | 0.0146 | No |
| 2 | PRDX4 |  |  | 41 | 0.455 | 0.0300 | No |
| 3 | HNRNPU |  |  | 71 | 0.373 | 0.0405 | No |
| 4 | HSP90AB1 |  |  | 74 | 0.371 | 0.0540 | No |
| 5 | KPNA2 |  |  | 157 | 0.308 | 0.0558 | No |
| 6 | DDX18 |  |  | 279 | 0.250 | 0.0510 | No |
| 7 | XPO1 |  |  | 390 | 0.220 | 0.0463 | No |
| 8 | SMARCC1 |  |  | 397 | 0.219 | 0.0537 | No |
| 9 | MCM4 |  |  | 482 | 0.201 | 0.0514 | No |
| 10 | RRM1 |  |  | 537 | 0.194 | 0.0522 | No |
| 11 | TUFM |  |  | 626 | 0.183 | 0.0488 | No |
| 12 | CCT5 |  |  | 746 | 0.165 | 0.0410 | No |
| 13 | PSMD1 |  |  | 748 | 0.165 | 0.0470 | No |
| 14 | NOP56 |  |  | 829 | 0.155 | 0.0434 | No |
| 15 | RFC4 |  |  | 835 | 0.154 | 0.0485 | No |
| 16 | MAD2L1 |  |  | 846 | 0.153 | 0.0530 | No |
| 17 | HNRNPA2B1 |  |  | 863 | 0.151 | 0.0568 | No |
| 18 | CCT2 |  |  | 878 | 0.150 | 0.0607 | No |
| 19 | MCM7 |  |  | 949 | 0.143 | 0.0578 | No |
| 20 | DHX15 |  |  | 1125 | 0.128 | 0.0421 | No |
| 21 | SF3B3 |  |  | 1183 | 0.123 | 0.0400 | No |
| 22 | CCT3 |  |  | 1198 | 0.122 | 0.0429 | No |
| 23 | PSMD14 |  |  | 1227 | 0.120 | 0.0441 | No |
| 24 | GSPT1 |  |  | 1324 | 0.114 | 0.0371 | No |
| 25 | SRSF7 |  |  | 1328 | 0.113 | 0.0409 | No |
| 26 | SSB |  |  | 1559 | 0.100 | 0.0178 | No |
| 27 | SRSF2 |  |  | 1697 | 0.092 | 0.0052 | No |
| 28 | MCM2 |  |  | 1762 | 0.088 | 0.0010 | No |
| 29 | EIF4A1 |  |  | 1767 | 0.088 | 0.0038 | No |
| 30 | DDX21 |  |  | 1824 | 0.085 | 0.0004 | No |
| 31 | EIF3B |  |  | 2209 | 0.068 | -0.0419 | No |
| 32 | MCM6 |  |  | 2237 | 0.067 | -0.0426 | No |
| 33 | KPNB1 |  |  | 2293 | 0.065 | -0.0466 | No |
| 34 | MYC |  |  | 2324 | 0.064 | -0.0478 | No |
| 35 | MCM5 |  |  | 2344 | 0.063 | -0.0477 | No |
| 36 | IFRD1 |  |  | 2444 | 0.059 | -0.0570 | No |
| 37 | SRSF1 |  |  | 2450 | 0.059 | -0.0555 | No |
| 38 | CCNA2 |  |  | 2529 | 0.056 | -0.0625 | No |
| 39 | NPM1 |  |  | 2533 | 0.056 | -0.0608 | No |
| 40 | PSMA4 |  |  | 2605 | 0.053 | -0.0671 | No |
| 41 | CDC45 |  |  | 2615 | 0.053 | -0.0662 | No |
| 42 | XRCC6 |  |  | 2637 | 0.052 | -0.0667 | No |
| 43 | HSPD1 |  |  | 2700 | 0.050 | -0.0721 | No |
| 44 | CTPS1 |  |  | 2789 | 0.047 | -0.0806 | No |
| 45 | PSMA2 |  |  | 3044 | 0.040 | -0.1088 | No |
| 46 | EIF2S1 |  |  | 3099 | 0.038 | -0.1137 | No |
| 47 | NCBP1 |  |  | 3108 | 0.038 | -0.1132 | No |
| 48 | CAD |  |  | 3181 | 0.036 | -0.1202 | No |
| 49 | TRIM28 |  |  | 3269 | 0.034 | -0.1291 | No |
| 50 | SRSF3 |  |  | 3283 | 0.034 | -0.1294 | No |
| 51 | CCT7 |  |  | 3307 | 0.033 | -0.1309 | No |
| 52 | EXOSC7 |  |  | 3309 | 0.033 | -0.1298 | No |
| 53 | PSMC4 |  |  | 3328 | 0.032 | -0.1307 | No |
| 54 | TARDBP |  |  | 3519 | 0.028 | -0.1518 | No |
| 55 | HNRNPA3 |  |  | 3532 | 0.027 | -0.1522 | No |
| 56 | ABCE1 |  |  | 3645 | 0.024 | -0.1644 | No |
| 57 | CCT4 |  |  | 3867 | 0.018 | -0.1895 | No |
| 58 | SNRPB2 |  |  | 3881 | 0.018 | -0.1904 | No |
| 59 | BUB3 |  |  | 3884 | 0.018 | -0.1899 | No |
| 60 | SNRPA1 |  |  | 3957 | 0.016 | -0.1978 | No |
| 61 | PCNA |  |  | 3986 | 0.016 | -0.2005 | No |
| 62 | CUL1 |  |  | 4057 | 0.014 | -0.2081 | No |
| 63 | GNL3 |  |  | 4103 | 0.012 | -0.2129 | No |
| 64 | ILF2 |  |  | 4112 | 0.012 | -0.2134 | No |
| 65 | PRPS2 |  |  | 4120 | 0.012 | -0.2138 | No |
| 66 | PABPC4 |  |  | 4149 | 0.011 | -0.2166 | No |
| 67 | PSMD3 |  |  | 4152 | 0.011 | -0.2165 | No |
| 68 | SF3A1 |  |  | 4187 | 0.010 | -0.2201 | No |
| 69 | SRPK1 |  |  | 4218 | 0.009 | -0.2232 | No |
| 70 | ORC2 |  |  | 4299 | 0.007 | -0.2323 | No |
| 71 | PA2G4 |  |  | 4363 | 0.005 | -0.2395 | No |
| 72 | GOT2 |  |  | 4434 | 0.003 | -0.2476 | No |
| 73 | APEX1 |  |  | 4599 | -0.001 | -0.2667 | No |
| 74 | RUVBL2 |  |  | 4658 | -0.003 | -0.2733 | No |
| 75 | SYNCRIP |  |  | 4713 | -0.004 | -0.2795 | No |
| 76 | G3BP1 |  |  | 4777 | -0.005 | -0.2867 | No |
| 77 | PWP1 |  |  | 4958 | -0.010 | -0.3073 | No |
| 78 | PSMC6 |  |  | 5139 | -0.014 | -0.3278 | No |
| 79 | CSTF2 |  |  | 5140 | -0.014 | -0.3273 | No |
| 80 | NCBP2 |  |  | 5356 | -0.020 | -0.3517 | No |
| 81 | EIF3D |  |  | 5505 | -0.024 | -0.3680 | No |
| 82 | XPOT |  |  | 5548 | -0.026 | -0.3720 | No |
| 83 | RRP9 |  |  | 5569 | -0.026 | -0.3734 | No |
| 84 | EIF3J |  |  | 5590 | -0.027 | -0.3747 | No |
| 85 | PRPF31 |  |  | 5611 | -0.028 | -0.3760 | No |
| 86 | VDAC1 |  |  | 5671 | -0.029 | -0.3818 | No |
| 87 | FBL |  |  | 5681 | -0.029 | -0.3818 | No |
| 88 | CDC20 |  |  | 5818 | -0.033 | -0.3964 | No |
| 89 | EIF4E |  |  | 5835 | -0.034 | -0.3970 | No |
| 90 | PHB |  |  | 5852 | -0.034 | -0.3976 | No |
| 91 | EIF4H |  |  | 5857 | -0.035 | -0.3968 | No |
| 92 | CDK2 |  |  | 5871 | -0.035 | -0.3970 | No |
| 93 | TOMM70 |  |  | 5941 | -0.037 | -0.4037 | No |
| 94 | USP1 |  |  | 5963 | -0.038 | -0.4048 | No |
| 95 | UBA2 |  |  | 6021 | -0.040 | -0.4099 | No |
| 96 | VDAC3 |  |  | 6079 | -0.042 | -0.4150 | No |
| 97 | NOLC1 |  |  | 6241 | -0.048 | -0.4321 | No |
| 98 | AIMP2 |  |  | 6394 | -0.052 | -0.4479 | No |
| 99 | RSL1D1 |  |  | 6425 | -0.054 | -0.4494 | No |
| 100 | CLNS1A |  |  | 6427 | -0.054 | -0.4475 | No |
| 101 | TRA2B |  |  | 6483 | -0.056 | -0.4519 | No |
| 102 | PSMA1 |  |  | 6529 | -0.057 | -0.4550 | No |
| 103 | HNRNPR |  |  | 6620 | -0.062 | -0.4632 | No |
| 104 | HNRNPC |  |  | 6643 | -0.063 | -0.4634 | No |
| 105 | HNRNPD |  |  | 6663 | -0.064 | -0.4633 | No |
| 106 | SERBP1 |  |  | 6758 | -0.069 | -0.4717 | No |
| 107 | ETF1 |  |  | 6805 | -0.071 | -0.4744 | No |
| 108 | RNPS1 |  |  | 6814 | -0.072 | -0.4727 | No |
| 109 | COPS5 |  |  | 6815 | -0.072 | -0.4700 | No |
| 110 | POLE3 |  |  | 6878 | -0.075 | -0.4745 | No |
| 111 | PSMB2 |  |  | 6885 | -0.075 | -0.4724 | No |
| 112 | HDAC2 |  |  | 6905 | -0.076 | -0.4718 | No |
| 113 | SNRPA |  |  | 7028 | -0.083 | -0.4830 | No |
| 114 | CBX3 |  |  | 7029 | -0.083 | -0.4799 | No |
| 115 | PGK1 |  |  | 7115 | -0.088 | -0.4866 | No |
| 116 | TCP1 |  |  | 7137 | -0.090 | -0.4857 | No |
| 117 | TFDP1 |  |  | 7144 | -0.090 | -0.4831 | No |
| 118 | STARD7 |  |  | 7220 | -0.095 | -0.4883 | No |
| 119 | MRPL9 |  |  | 7221 | -0.095 | -0.4848 | No |
| 120 | IMPDH2 |  |  | 7333 | -0.103 | -0.4939 | No |
| 121 | NOP16 |  |  | 7350 | -0.104 | -0.4919 | No |
| 122 | UBE2E1 |  |  | 7368 | -0.106 | -0.4900 | No |
| 123 | SNRPD1 |  |  | 7463 | -0.113 | -0.4967 | Yes |
| 124 | EEF1B2 |  |  | 7497 | -0.117 | -0.4963 | Yes |
| 125 | PSMD8 |  |  | 7500 | -0.117 | -0.4922 | Yes |
| 126 | NAP1L1 |  |  | 7538 | -0.121 | -0.4920 | Yes |
| 127 | SLC25A3 |  |  | 7543 | -0.122 | -0.4879 | Yes |
| 128 | RAD23B |  |  | 7601 | -0.128 | -0.4899 | Yes |
| 129 | PSMB3 |  |  | 7602 | -0.128 | -0.4851 | Yes |
| 130 | DUT |  |  | 7608 | -0.128 | -0.4809 | Yes |
| 131 | FAM120A |  |  | 7624 | -0.130 | -0.4779 | Yes |
| 132 | MRPS18B |  |  | 7626 | -0.130 | -0.4732 | Yes |
| 133 | YWHAE |  |  | 7628 | -0.131 | -0.4684 | Yes |
| 134 | SNRPD3 |  |  | 7648 | -0.132 | -0.4658 | Yes |
| 135 | PRDX3 |  |  | 7649 | -0.132 | -0.4608 | Yes |
| 136 | HPRT1 |  |  | 7679 | -0.137 | -0.4592 | Yes |
| 137 | EIF1AX |  |  | 7689 | -0.138 | -0.4551 | Yes |
| 138 | EIF4G2 |  |  | 7707 | -0.140 | -0.4519 | Yes |
| 139 | C1QBP |  |  | 7717 | -0.141 | -0.4477 | Yes |
| 140 | EIF2S2 |  |  | 7758 | -0.147 | -0.4469 | Yes |
| 141 | MRPL23 |  |  | 7776 | -0.148 | -0.4434 | Yes |
| 142 | TYMS |  |  | 7792 | -0.150 | -0.4395 | Yes |
| 143 | NME1 |  |  | 7811 | -0.154 | -0.4360 | Yes |
| 144 | CYC1 |  |  | 7816 | -0.154 | -0.4307 | Yes |
| 145 | GLO1 |  |  | 7844 | -0.157 | -0.4280 | Yes |
| 146 | NDUFAB1 |  |  | 7858 | -0.160 | -0.4236 | Yes |
| 147 | VBP1 |  |  | 7866 | -0.161 | -0.4185 | Yes |
| 148 | ERH |  |  | 7879 | -0.163 | -0.4138 | Yes |
| 149 | HSPE1 |  |  | 7918 | -0.170 | -0.4120 | Yes |
| 150 | PABPC1 |  |  | 7960 | -0.179 | -0.4101 | Yes |
| 151 | PSMD7 |  |  | 7976 | -0.181 | -0.4052 | Yes |
| 152 | RAN |  |  | 7979 | -0.181 | -0.3987 | Yes |
| 153 | UBE2L3 |  |  | 8045 | -0.192 | -0.3991 | Yes |
| 154 | PPM1G |  |  | 8072 | -0.198 | -0.3948 | Yes |
| 155 | ACP1 |  |  | 8085 | -0.201 | -0.3887 | Yes |
| 156 | PHB2 |  |  | 8125 | -0.210 | -0.3855 | Yes |
| 157 | RANBP1 |  |  | 8132 | -0.212 | -0.3783 | Yes |
| 158 | PCBP1 |  |  | 8153 | -0.216 | -0.3727 | Yes |
| 159 | CDK4 |  |  | 8168 | -0.220 | -0.3661 | Yes |
| 160 | SET |  |  | 8175 | -0.221 | -0.3587 | Yes |
| 161 | AP3S1 |  |  | 8213 | -0.232 | -0.3544 | Yes |
| 162 | HDGF |  |  | 8267 | -0.246 | -0.3514 | Yes |
| 163 | PSMA7 |  |  | 8288 | -0.255 | -0.3443 | Yes |
| 164 | YWHAQ |  |  | 8360 | -0.278 | -0.3423 | Yes |
| 165 | HDDC2 |  |  | 8374 | -0.282 | -0.3333 | Yes |
| 166 | DEK |  |  | 8376 | -0.284 | -0.3229 | Yes |
| 167 | CNBP |  |  | 8379 | -0.286 | -0.3125 | Yes |
| 168 | RPS10 |  |  | 8404 | -0.297 | -0.3043 | Yes |
| 169 | RACK1 |  |  | 8406 | -0.297 | -0.2934 | Yes |
| 170 | RPL18 |  |  | 8424 | -0.304 | -0.2841 | Yes |
| 171 | SNRPG |  |  | 8443 | -0.312 | -0.2746 | Yes |
| 172 | RPL14 |  |  | 8458 | -0.320 | -0.2644 | Yes |
| 173 | LSM7 |  |  | 8471 | -0.325 | -0.2537 | Yes |
| 174 | COX5A |  |  | 8481 | -0.331 | -0.2425 | Yes |
| 175 | POLD2 |  |  | 8549 | -0.363 | -0.2368 | Yes |
| 176 | LSM2 |  |  | 8558 | -0.371 | -0.2240 | Yes |
| 177 | ODC1 |  |  | 8576 | -0.385 | -0.2117 | Yes |
| 178 | SNRPD2 |  |  | 8579 | -0.386 | -0.1976 | Yes |
| 179 | RPS3 |  |  | 8598 | -0.400 | -0.1849 | Yes |
| 180 | RPL22 |  |  | 8613 | -0.416 | -0.1711 | Yes |
| 181 | PPIA |  |  | 8626 | -0.432 | -0.1565 | Yes |
| 182 | NHP2 |  |  | 8632 | -0.436 | -0.1408 | Yes |
| 183 | TXNL4A |  |  | 8635 | -0.440 | -0.1247 | Yes |
| 184 | RPS5 |  |  | 8656 | -0.460 | -0.1100 | Yes |
| 185 | RPL34 |  |  | 8671 | -0.476 | -0.0940 | Yes |
| 186 | RPL6 |  |  | 8688 | -0.506 | -0.0771 | Yes |
| 187 | SRM |  |  | 8693 | -0.511 | -0.0586 | Yes |
| 188 | RPLP0 |  |  | 8703 | -0.524 | -0.0402 | Yes |
| 189 | RPS6 |  |  | 8718 | -0.559 | -0.0211 | Yes |
| 190 | RPS2 |  |  | 8739 | -0.668 | 0.0014 | Yes |
Table: GSEA details [plain text format]

  

Fig 2: HALLMARK\_MYC\_TARGETS\_V1: Random ES distribution      
 Gene set null distribution of ES for **HALLMARK\_MYC\_TARGETS\_V1**
